# Microfluidic platform for three-dimensional cell culture under spatiotemporal heterogeneity of oxygen tension

**DOI:** 10.1063/1.5127069

**Published:** 2020-03-06

**Authors:** Rei Koens, Yugo Tabata, Jean C. Serrano, Satoshi Aratake, Daisuke Yoshino, Roger D. Kamm, Kenichi Funamoto

**Affiliations:** 1 Graduate School of Biomedical Engineering, Tohoku University, 6-6-12 Aramaki-aza Aoba, Aoba-ku, Sendai, Miyagi 980-8579, Japan; 2 Institute of Fluid Science, Tohoku University, 2-1-1 Katahira, Aoba-ku, Sendai, Miyagi 980-8577, Japan; 3 Department of Mechanical Engineering, Massachusetts Institute of Technology, 77 Massachusetts Ave., Cambridge, Massachusetts 02139, USA; 4 School of Engineering, Tohoku University, 6-6, Aramaki-aza Aoba, Aoba-ku, Sendai, Miyagi 980-8579, Japan; 5 Frontier Research Institute for Interdisciplinary Sciences, Tohoku University, 6-3 Aramaki-aza Aoba, Aoba-ku, Sendai, Miyagi 980-8578, Japan; 6 Institute of Engineering, Tokyo University of Agriculture and Technology, 2-24-16 Nakacho, Koganei, Tokyo 184-8588, Japan

## Abstract

Cells in a tumor microenvironment are exposed to spatial and temporal variations in oxygen tension due to hyperproliferation and immature vascularization. Such spatiotemporal oxygen heterogeneity affects the behavior of cancer cells, leading to cancer growth and metastasis, and thus, it is essential to clarify the cellular responses of cancer cells to oxygen tension. Herein, we describe a new double-layer microfluidic device allowing the control of oxygen tension and the behavior of cancer cells under spatiotemporal oxygen heterogeneity. Two parallel gas channels were located above the media and gel channels to enhance gas exchange, and a gas-impermeable polycarbonate film was embedded in the device to prevent the diffusion of atmospheric oxygen. Variations in oxygen tension in the device with the experimental parameters and design variables were investigated computationally and validated by using oxygen-sensitive nanoparticles. The present device can generate a uniform hypoxic condition at oxygen levels down to 0.3% O_2_, as well as a linear oxygen gradient from 3% O_2_ to 17% O_2_ across the gel channel within 15 min. Moreover, human breast cancer cells suspended in type I collagen gel were introduced in the gel channel to observe their response under controlled oxygen tension. Hypoxic exposure activated the proliferation and motility of the cells, which showed a local maximum increase at 5% O_2_. Under the oxygen gradient condition, the increase in the cell number was relatively high in the central mild hypoxia region. These findings demonstrate the utility of the present device to study cellular responses in an oxygen-controlled microenvironment.

## INTRODUCTION

Low oxygen tension (hypoxia) is a major stimulus affecting cell behavior and cell–cell interactions^1^ and is commonly found in both physiological and pathological conditions. *In vivo* oxygen tension is usually expressed as a percentage of the atmospheric oxygen concentration (21%) and is below 10% O_2_ even in healthy organs.^2^ Pathological events can further decrease oxygen concentration to levels below 1%, as observed in tumor microenvironments.^3^ The hyperproliferation of cells and immature vascularization in the tumor microenvironment lead to a hypoxic condition with a non-uniform spatial distribution of oxygen tension^4^ and a temporal variation of oxygen tension due to ischemia and reperfusion.^5,6^ Consequently, the cells and extracellular matrix (ECM) are exposed to a spatiotemporal heterogeneity of oxygen tension.^7^ Hypoxic microenvironments allow cancer cells to proliferate at the primary site and disseminate, invading the surrounding tissues, blood vessels, and lymph vessels and metastasizing toward secondary sites.^8,9^ Cancer cell migration depends on mechanical stimuli, such as interstitial flow^10,11^ and ECM stiffness,^12^ and chemical stimuli.^13,14^ Hypoxic stress represents a chemical signal that can alter cancer cell metabolism and activity related to proliferation, invasion, and migration.^15–20^ Acute, cyclic hypoxic stress enhances tumor metastasis.^21,22^ In addition, hypoxic conditions in the tumor microenvironment induce resistance to anti-cancer drugs.^23,24^ It is therefore important to understand cancer cell responses to a wide range of oxygen tensions with different spatiotemporal variations,^25,26^ but currently, little is known regarding the oxygen dependency of cancer cell behavior.

The effects of hypoxic exposure on cells have been investigated by various methods.^27,28^ Conventional assays utilize a gas-controlled incubator or glovebox, but it is difficult to control spatiotemporal oxygen tension in microenvironments using this approach. In contrast, microfluidic techniques are promising for precisely controlling oxygen tension during cellular experiments. Microfluidic devices made of gas-permeable poly(dimethylsiloxane) (PDMS) are widely used for cellular experiments^29^ and enable three-dimensional (3D) cell culture, high-resolution live-cell imaging, precise control over mechanical and chemical stimuli to the cells,^30^ and reproduction of the *in vivo* microenvironment.^31^ A variety of microfluidic devices, called either tumor-on-a-chip or cancer-on-a-chip, mimic the tumor microenvironment and have been proposed as devices to study the process of cancer progression and metastasis.^9,32,33^ Microfluidic devices allowing the control of oxygen tension have been used to study the effects of oxygen tension on cells and oxygen consumption by cells.^27,28,34^ Oxygen tension inside such devices is generally achieved using chemical reactions,^35–38^ by introducing gas mixtures with a predefined oxygen concentration into the microchannels,^39,40^ or by using gas exchanger bottles.^41^ The authors previously developed a single-layer microfluidic device to observe cell behavior in a 3D space under controlled oxygen tensions.^42^ A uniform oxygen tension or an oxygen gradient was generated in the device by supplying gas mixtures to the gas channels flanking the media and gel channels and by embedding a polycarbonate (PC) film with low gas permeability above the channels. The device was then used for hypoxic cell experiments quantifying the migration of breast cancer cells^42^ and the permeability of endothelial monolayers.^43^ However, the lowest oxygen level possible using this approach was about 3% O_2_ and over an hour was required to establish a steady-state oxygen level because of limited gas exchange between the channels when placed on the same horizontal plane. For instance, the oxygen level in a tumor microenvironment falls below 3% O_2_ and can vary on a time scale of minutes.^3,6^ Hence, an ideal system for controlling oxygen tension in a microfluidic device would allow the generation and regulation of an arbitrary oxygen tension down to 0% within minutes.

**FIG. 1.**
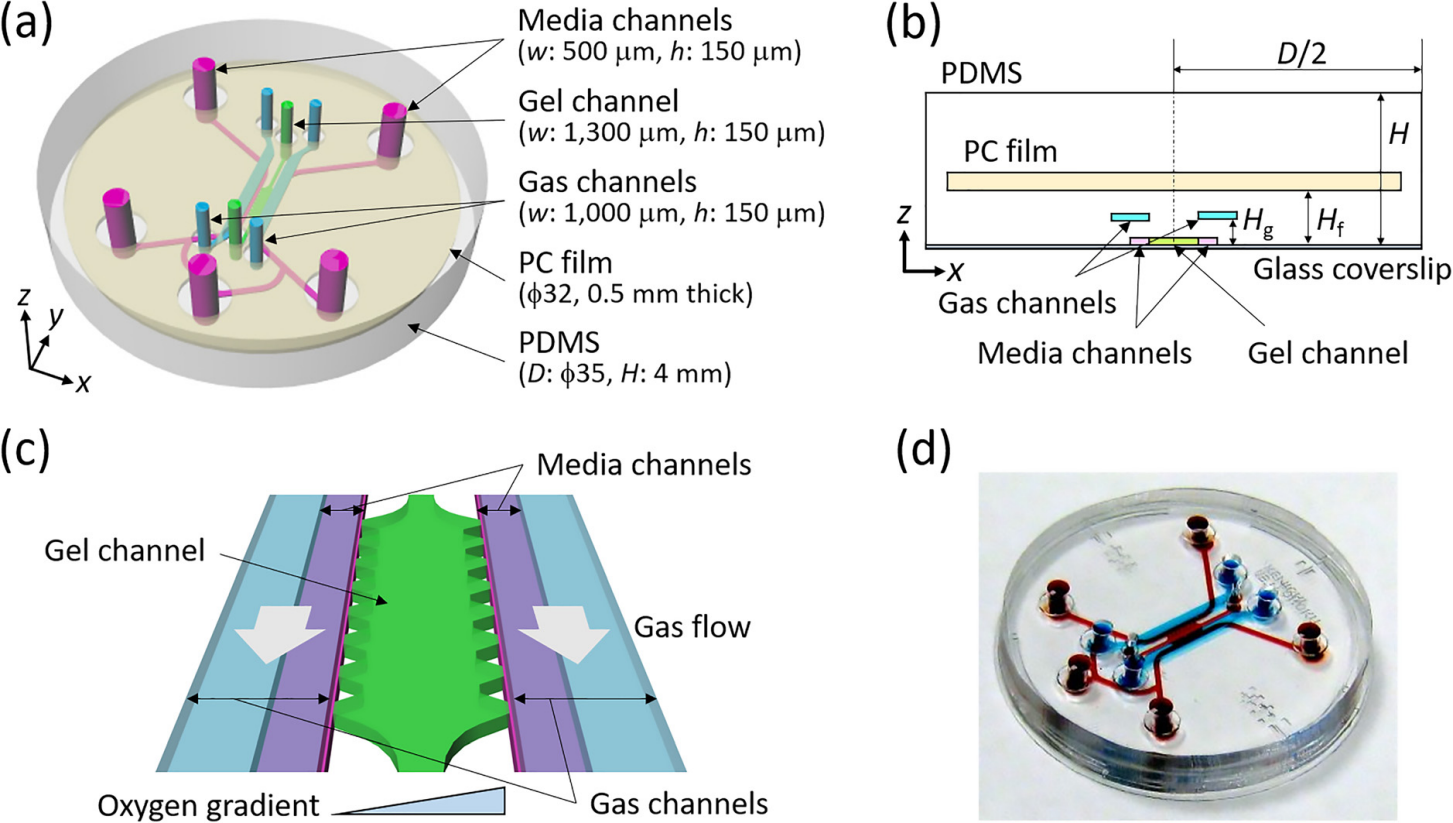
Design of the present double-layer microfluidic device: (a) an overhead view, with definitions of the coordinates, (b) a cross-sectional view (the *zx*-plane) at *y *=* *0.2 mm, and (c) an enlarged view of the cell culture region. Supplying gas mixtures to the two separated gas channels located at *H*_g_ above the media and gel channels allows control of the oxygen tension inside the device. A polycarbonate (PC) film is embedded at *H*_f_ inside the microfluidic device to prevent oxygen diffusion from the atmosphere. (d) A photograph of the actual device. Red ink fills the media and gel channels, and blue ink fills the gas channels.

In this study, we improved our previously reported microfluidic device to overcome limitations in reproducing *in vivo* spatiotemporally heterogeneous oxygen tensions. The device was modified to be a double-layer system by repositioning the gas channels above the media channels to promote gas exchange between the channels while retaining the embedded gas-impermeable PC film (Fig. 1). The experimental parameters and design variables were optimized by numerical simulations to enhance the control of oxygen tension. Oxygen tensions generated in the device using appropriate settings were then validated using oxygen-sensitive nanoparticles embedded in the gel channel. The computational and experimental results demonstrate that the present device controls oxygen tension more rapidly (≤15 min) and generates a lower oxygen level (as low as 0.3% O_2_) than the previous device. The feasibility of using this device for cellular experiments was examined by investigating the behavior of human breast cancer cells (MDA-MB-231 cell line) under spatially or temporally controlled oxygen tension. We found that the cells are activated after hypoxic exposure, promoting their migration and proliferation, and that the cells tend to migrate toward an area of mild hypoxia when cultured under an oxygen gradient.

## RESULTS AND DISCUSSION

### Optimization of system parameters for controlling oxygen tension

Numerical simulations revealed changes in oxygen tension in the microfluidic device as a function of the experimental parameters and design variables. Gas mixtures supplied at a relatively low flow rate, i.e., *Pe*_g_ > 10 (*Q*_g_ > 1.8 ml/min), allowed generation of a constant oxygen condition in the media and gel channels (Fig. S1). In contrast, the flow rate of the medium significantly affected the oxygen tension because the medium flowed away prior to the oxygen tension equilibrating at high flow rates (Fig. S2). A medium flow rate of *Pe_m_* < 100 (*Q*_m_ < 1.8 × 10^−3 ^ml/min) was required to maintain a constant oxygen level. These results concerning flow rates were consistent with those observed using the former single-layer device.^42^ Hereafter, *Pe*_g_ = 100 (*Q*_g_ = 18 ml/min) was used as the optimal gas supply flow rate and no medium flow (*Pe*_m_ = 0) was applied to simplify the experimental setup.

**FIG. 2.**
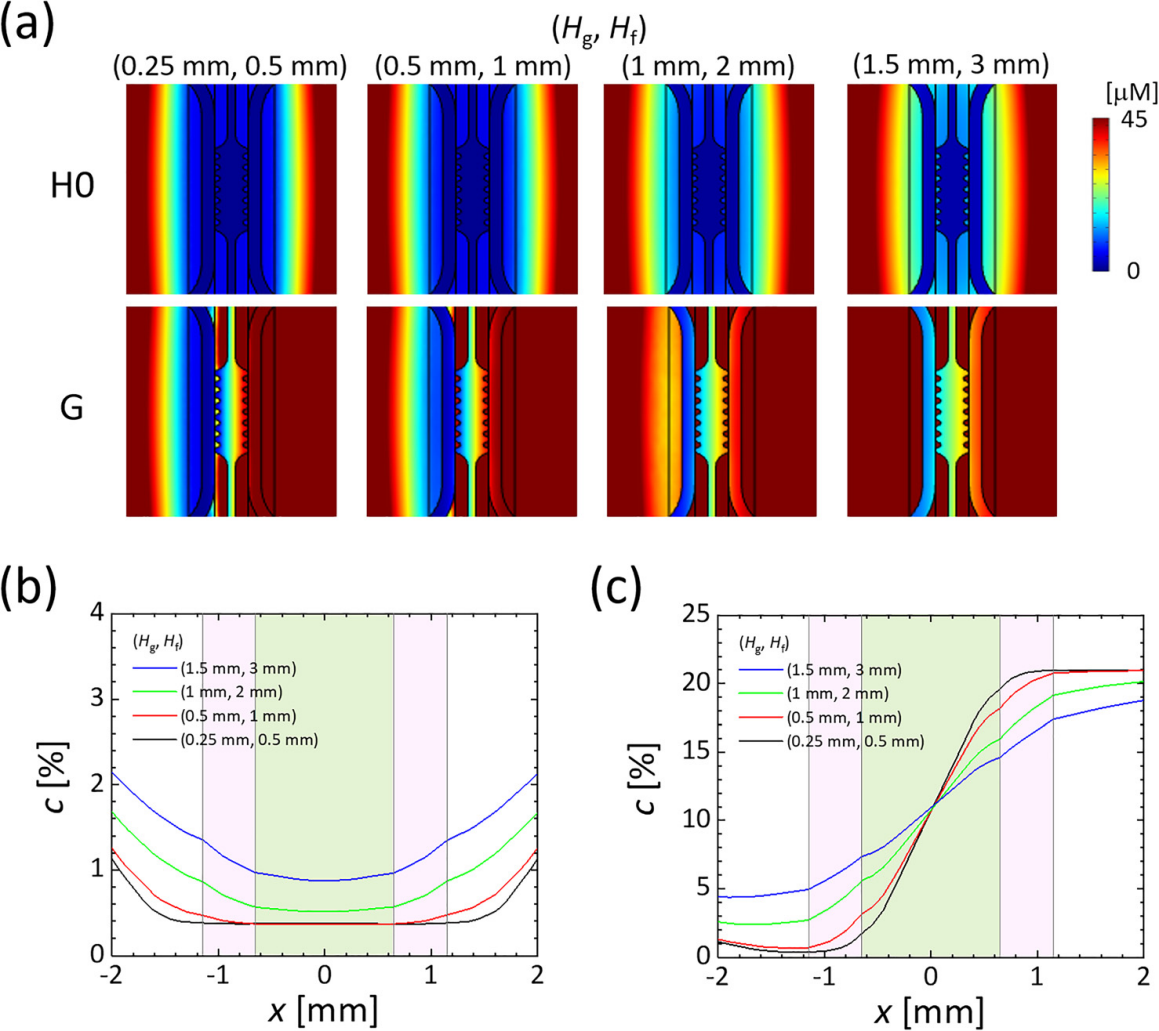
Computational results of steady oxygen tension in the microfluidic device as the locations of the gas channels and PC film (*H*_g_, *H*_f_) were varied. Péclet numbers for the medium and gas flows were set at (*Pe*_m_, *Pe*_g_) = (0, 100). (a) Oxygen distributions on a horizontal cross section (*z *=* *0 mm), generated by supplying a gas mixture containing 0% O_2_ to both gas channels (H0) or by supplying gas mixtures with 0% and 21% O_2_ to the left- and right-hand gas channels, respectively (G). Oxygen tension profiles across the gel channel (*y *=* *0.2 mm) under the oxygen conditions of (b) H0 and (c) G. The origin was set at the center of the gel channel, and the *x*-direction was defined as the horizontal direction normal to the gel channel. Regions shaded with pink and green indicate sections of the media and gel channels, respectively.

The locations (*H*_g_, *H*_f_) of the gas channels and PC film are critical factors affecting the oxygen tension inside the device. The oxygen concentration in the media and gel channels increased as the gas channels and PC film were placed higher above the media and gel channels [Fig. 2(a)]. This is likely due to an increase in the oxygen leakage from the sides of the device, thus reducing the ability of the low oxygen in the channels to compete against the contribution of oxygen diffusion from the side walls. The PC film needed to be located at a height of less than 2 mm to generate a uniform oxygen tension of less than 1% O_2_ in the media and gel channels [Fig. 2(b)]. Without the PC film, the oxygen tension showed a convex profile in the gel channel due to oxygen diffusion from the top surface even if the width of the gas channels was enlarged (Fig. S3). The oxygen tension gradient was also dependent on the height of the gas channel *H*_g_ [Fig. 2(c)]: the lower the height, the steeper the gradient. Thus, fabrication of these devices requires that the thickness of each PDMS layer of the media and gel channels, and of the gas channels, is kept less than or equal to 0.5 mm to allow precise control of the oxygen tension. It should be noted that it became technically difficult to transfer the media and gel microchannel pattern to a thin PDMS layer, during device fabrication, in cases that the thickness *H*_g_ was less than 0.5 mm. In summary, using the design parameters (*H*_g_, *H*_f_) = (0.5 mm, 1 mm), numerical simulations of steady-state oxygen tension indicated that a uniform oxygen tension of 0.4% O_2_, as well as a steep linear oxygen gradient across the gel from 3% O_2_ to 18% O_2_, can be established in the current device.

We also analyzed the transient states of oxygen tension after switching the gas mixtures supplied to the gas channels. When a gas mixture with 0% O_2_ was supplied to both gas channels in the device under a normoxic atmosphere, the hypoxic region reached a uniform state after 10 min at the center of the device (Fig. S4). Similarly, when the supplied gas mixture was changed from 0% O_2_ to 21% O_2_, the device was reoxygenated to the normoxic state within 10 min (Fig. S5).

### Validation of oxygen tension using phosphorescence intensity

**FIG. 3.**
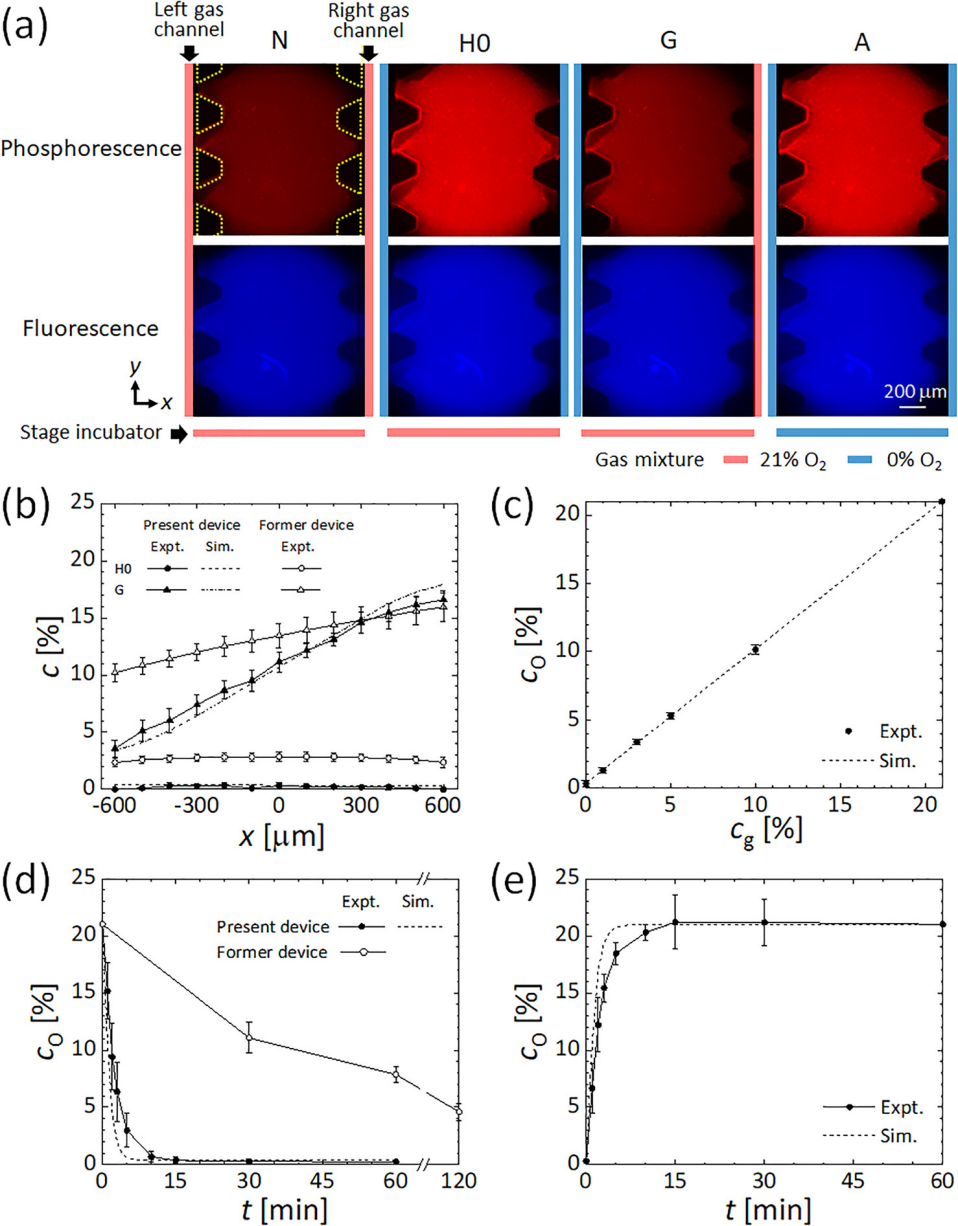
Results of the validation experiments: (a) microscope images of phosphorescence and fluorescence from the oxygen-sensitive nanoparticles immobilized in type I collagen gel under oxygen conditions of normoxia (N), hypoxia (H0) generated by supplying a gas mixture with 0% O_2_ to both gas channels while supplying a gas mixture with 21% O_2_ to the stage incubator, an oxygen gradient (G) generated by supplying gas mixtures with 0% and 21% O_2_ to the left-hand and right-hand side gas channels, respectively, while supplying a gas mixture with 21% O_2_ to the stage incubator, and anoxia (A) generated by supplying a gas mixture with 0% O_2_ to both gas channels and the stage incubator. Colored bars adjacent to each image represent the oxygen concentration in the gas mixture supplied to the left and right gas channels and the stage incubator. Yellow dotted trapezoids indicate the PDMS pillars supporting the media and gel channels. (b) Comparison of the steady oxygen tension profile across the gel channel between the present double-layer and former single-layer devices^42^ for the conditions H0 and G. The present device can generate a lower uniform oxygen condition and a steeper oxygen gradient than the former device. (c) Linear variation of the steady oxygen tension *c*_O_ at the center of the gel channel (*x *=* *0 mm) with the oxygen concentration *c*_g_ in the gas mixture supplied to both gas channels. Transient oxygen tensions at the center of the gel channel after switching the oxygen conditions (d) from N to H0 and (e) vice versa. The oxygen tension equilibrates within 15 min of the switch.

The intensity of red phosphorescence of oxygen-sensitive nanoparticles embedded in the gel channel was oxygen-dependent [Fig. 3(a)], with brighter images observed under lower oxygen tensions. In contrast, the blue fluorescence intensity of the particles remained constant regardless of the oxygen tension, indicating that no obvious photobleaching occurred. Steady-state profiles of oxygen tension as obtained from the intensity of phosphorescence across the gel channel were compared with the computational results [Fig. 3(b)]. The oxygen condition H0, obtained by supplying a 0% O_2_ gas mixture to both gas channels, generated a uniform oxygen tension of 0.3% O_2_. Oxygen gradient condition G, obtained by supplying gas mixtures with 0% and 21% O_2_ to the left-hand and right-hand side gas channels, respectively, generated a linear oxygen gradient from 3% O_2_ to 17% O_2_ across the gel channel. Both measurements agreed well with the corresponding computational results although a small difference of 1% O_2_ was observed between the measurement and computational results on the right-hand side of the gel channel in the oxygen gradient case, perhaps due to variation in the locations of the gas channels and PC film and misalignment of the gas channels to the other channels. In comparison with the former single-layer device,^42^ the present double-layer device generated a lower uniform oxygen tension and a steeper oxygen gradient.

Uniform oxygen tension could be controlled to a level as low as 0.3% by changing the oxygen concentration *c*_g_ in the gas mixture supplied to both gas channels (Fig. S6). The oxygen tension *c*_O_ at the center (*x *=* *0 mm) of the gel channel changed linearly with the oxygen concentration *c*_g_ in the gas mixture supplied to the gas channels, in good agreement with the computational result [Fig. 3(c)]. An oxygen concentration *c*_g_ in the gas mixture of 0%, 1%, 3%, 5%, and 10% resulted in *c*_O_ values of 0.3%, 1.3%, 3.4%, 5.3%, and 10.1%, respectively. Thus, the oxygen tension inside the device can be controlled at levels ranging from severe hypoxic conditions to normoxic conditions.

Transient changes in oxygen tension at the center of the gel channel, from normoxia (N) to hypoxia (H0) or vice versa, are shown in Figs. 3(d) and 3(e), in addition to a comparison of the corresponding computational results and measured data for oxygen tension in the former device.^42^ Oxygen tension steeply decreased after supplying a gas mixture with 0% O_2_ and reached a steady oxygen tension of 0.3% O_2_ within 15 min. Oxygen tension during reoxygenation from the hypoxic condition H0 rapidly increased over 10 min. The variation in time response between the measured oxygen tension and the computational results may be attributable to some uncertainty in the material parameters, or to non-ideal device fabrication and setup errors, resulting in the diffusion of atmospheric oxygen or mixing of residual former gas components in tubing, flowmeters, and humidifier bottles. Nevertheless, the present double-layer microfluidic device can generate a lower oxygen tension at a much faster rate than the former single-layer device.

### Behavior of breast cancer cells under uniform oxygen tensions

MDA-MB-231 human breast cancer cells seeded in collagen gel were observed under controlled oxygen tensions to investigate their oxygen-dependent behavior. Oxygen tensions generated in the device ranging between 0.3% and 21% O_2_ did not affect cell viability (Fig. S7). Cell viability exceeded 90% after 24 h of cell culture under all oxygen conditions with no significant differences between the oxygen conditions, consistent with earlier results.^44^ Also, we confirmed that there was no significant change in cell viability in the devices whether or not gas mixtures were supplied to the gas channels, implying that gas flow in the gas channels has no adverse effect on the culture environment.

**FIG. 4.**
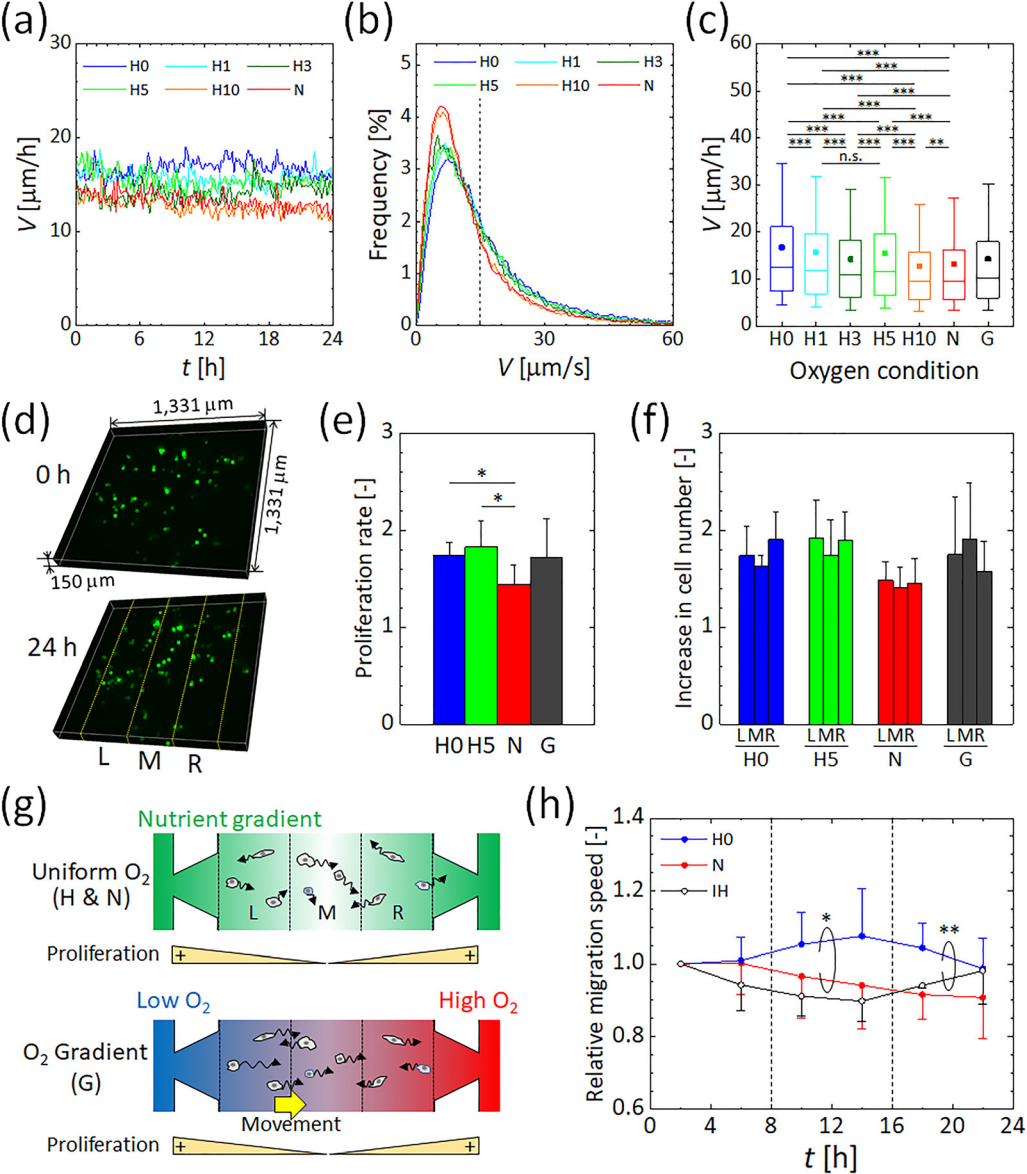
Behavior of MDA-MB-231 human breast cancer cells under uniform oxygen tensions and under an oxygen gradient. (a) Variations in the migration speed of the cells measured every 10 min under controlled oxygen tensions. Hypoxic conditions and normoxic conditions generated by supplying gas mixtures containing 0%–10% and 21% O_2_ to both gas channels are represented as H0–H10 and N, respectively. The oxygen tensions in the gel under the H0–H10 and N conditions were uniform at 0.3, 1.3, 3.4, 5.3, 10.1, and 21% O_2_. (b) Frequency distribution for the migration speed of the cells under uniform oxygen conditions H0–H10 and N. The vertical dashed line indicates the threshold to divide the cells into two groups: cells with slow and fast migration speed. (c) Box-and-whisker plots of the migration speed of the cells. The upper and lower extremes represent the 90th and 10th percentiles, the box plot represents quartiles, and the band and dot inside each box show the median and the average value, respectively. The oxygen gradient G was generated by supplying gas mixtures containing 0% and 21% O_2_ to the left and right gas channels, respectively. The oxygen tension generated under condition G had a linear oxygen gradient from 3% O_2_ (left) to 17% O_2_ (right) across the gel. Significant changes in the migration speed of the cancer cells by oxygen concentration were assessed by the Kruskal–Wallis tests followed by Dunn's post-hoc tests for multiple comparisons. ^**^p <* *0.01; ^***^p <* *0.001; n.s., not significant. (d) Representative 3D distributions of the cells in the collagen gel before and after 24-h exposure to normoxic condition N. Yellow dotted lines indicate the boundaries of three equivalent regions L, M, and R divided in the *x*-direction, used to evaluate the increase in the cell number. (e) Proliferation rate of the cells in the gel channel and (f) increase in the cell number in the three equivalent regions after 24 h under the oxygen conditions H0, H5, N, and G. (g) Schematic showing cancer cell migration under the uniform oxygen conditions H and N or along an oxygen gradient G. Color shading indicates the nutrient concentration or oxygen concentration in the media and gel channels. The higher nutrient region is colored with thicker green. The oxygen tension in the blue region is lower than that in the red region. (h) Relative migration speed averaged every 4 h under the steady oxygen conditions H0 and N and the intermittent hypoxic condition IH, obtained by normalizing each result to the first time period. The IH condition was generated by changing the oxygen conditions inside the device from N to H0 and returning to N at 8 h intervals. Significant differences between variations of migration speed under different oxygen conditions were assessed by the two-way ANOVA test. ^*^p <* *0.05; ^**^p <* *0.01. Error bars in (e), (f), and (h) represent the standard deviation.

3D migration trajectories of the breast cancer cells in the gel channel were tracked by detecting their centroid (Fig. S8). Under uniformly controlled oxygen tensions, the average migration speed of the cells in 3D ECM at each time point remained essentially constant over 24 h [Fig. 4(a)] and these values were consistent with earlier findings.^10–12,14^ The lower the oxygen concentration, the faster the migration speed tended to be. The slight decrease in migration speed observed with time might be caused by the consumption of nutrients in the medium or by cell damage resulting from exposure to the excitation light required for confocal imaging. The distribution of migration speeds for cells under each uniform oxygen tension (H0–H10 and N) is represented by a frequency distribution and a boxplot diagram in Figs. 4(b) and 4(c), respectively. Oxygen-dependent cell migration can be clearly observed, with faster migration under hypoxia than under normoxia conditions. The cells could be divided into two groups by setting a threshold for the cell migration velocity at around 15 *μ*m/h, which is approximately twofold the migration speed where the peak of frequency distribution was observed, in each oxygen condition [Fig. 4(b)]. As the oxygen tension decreased, there was no remarkable change in the migration speed at the peak of frequency distribution in the slowly migrating subpopulation, while the migration speed in the fast migrating subpopulation increased. The average value of the migration speed was higher than the median value for each oxygen condition [Fig. 4(c)], indicating that a relatively small number of rapidly migrating cells increased their relative average value. Kruskal–Wallis tests performed on the cell migration speeds revealed significant differences among the uniform oxygen conditions (*p *<* *0.001). Furthermore, multiple comparisons by Dunn's post-hoc tests indicated that hypoxic conditions significantly increased the migration speed from the normoxic condition N [Fig. 4(c)]. Significant differences were also found between the hypoxic conditions except for between the hypoxic conditions H1 and H5. These results suggest that a decrease in oxygen tension stimulates cells to migrate, especially for those in an active state to migrate. Previous studies have also reported an increase in the invasiveness of cancer cells under hypoxia.^15–18,45,46^ Interestingly, the migration speed showed a local maximum value under oxygen condition H5 (oxygen tension generated at 5% O_2_) [Fig. 4(c)]. In contrast, the migration speed was almost the same under oxygen conditions H10 and N (oxygen tensions generated by 10% and 21% O_2_, respectively). We conclude that MDA-MB-231 cells respond to distinctive oxygen conditions that stimulate them to actively migrate.

### Cell proliferation under controlled oxygen tensions

Cell proliferation rates were measured over a 24 h period by counting the number of cells before and after the experiment [Fig. 4(d)] under the following conditions: H0 and H5 (0.3% and 5% O_2_ concentration), the normoxic condition N, and the oxygen gradient condition G [a linear oxygen gradient from 3% O_2_ (left) to 17% O_2_ (right) inside the gel] [Fig. 4(e)]. Hypoxic conditions significantly promoted proliferation of MDA-MB-231 cells, which increased 1.7 and 1.8-fold under the hypoxic conditions H0 and H5, respectively, after 24 h, compared to a 1.4-fold increase under the normoxic condition N. This enhanced proliferation under hypoxia is in agreement with an earlier report that MDA-MB-231 cells proliferated more after a day of hypoxic exposure (1% O_2_) than under normoxia.^22^ However, this result differs from the results obtained following long-term cell culture for more than two days,^17,20^ perhaps due to the 3D cell culture conditions and culture period used in the current study. Under oxygen gradient G, the cells proliferated 1.7-fold, similar to the proliferation rates in uniform normoxic and hypoxic environments.

### Directional migration of cancer cells in an oxygen tension gradient

Migration speed of the cells under an oxygen gradient was intermediate between that under either normoxic or hypoxic conditions [Fig. 4(c)]. To assess directional cell migration, the gel channel (−450 *μ*m ≤ *x *≤* *450 *μ*m) was divided into three equivalent regions in the *x*-direction [see Figs. 4(d) and 4(g)], and the increase in cell number over a 24 h period was determined under each condition [Fig. 4(f)]. In all oxygen conditions, the increase in the cell number between the three equivalent regions L, M, and R did not exhibit statistically significant differences. But the increase in the cell number tended to be higher in the outer regions than in the central region under uniform oxygen conditions, especially under hypoxic conditions. The cells did not show notable movements toward the outer regions of the gel channel under those uniform oxygen conditions (Fig. S9). Hence, the different increase in the cell number could be attributable to proliferation under a nutrient concentration gradient between the central region and the outer regions; the cells were proliferated more in the nutrient-rich regions L and R adjacent to the media channels [Fig. 4(g)].^47^ In contrast to the uniform oxygen conditions, this increase was relatively high in the central region M under an oxygen gradient and was relatively low in the right-hand side region R where the oxygen concentration is the highest [Fig. 4(f)]. In the left and middle regions, the percentage of the cells with the positive *x*-directional migration velocity was larger than the opposite one (Fig. S9). To further investigate the directional migration of the cells in the oxygen tension gradient, an additional cellular experiment was conducted under different oxygen gradient G2 in a lower oxygen range than condition G, by supplying gas mixtures at 0% and 10% O_2_ to the left-hand and right-hand side gas channels, respectively (Fig. S10). The numerical simulation of oxygen tension showed that the experimental condition generated an oxygen gradient ranging from 2% to 9% O_2_ across the gel channel. Similar to the oxygen gradient condition G, the increase in the cell number under the condition G2 showed a distribution different from the uniform oxygen conditions. There are conflicting reports for MDA-MB-231 cell migration under oxygen gradient conditions, with some reports of cells migrating toward the higher oxygen side^48^ and some toward the lower oxygen side.^38^ Given the present findings that both the proliferation and migration of MDA-MB-231 cells were promoted in hypoxic conditions, there is a possibility that the cells were activated under low oxygen conditions and migrated toward an area of mild hypoxia between 5% and 10% O_2_ [Fig. 4(g)]. However, the observed phenomenon might also be the consequence of nutrient/metabolite gradients generated across the gel channel, and thus, further experiments are required.

### Migration change by temporal heterogeneity of oxygen tension

We also examined the effect of intermittent hypoxia on MDA-MB-231 cells. The migration speed of the cells was compared under the steady hypoxic and normoxic conditions H0 and N and the intermittent hypoxic condition IH. Average migration speeds were calculated every 4 h, and the results were normalized by the value obtained after the first 4 h [Fig. 4(h)]. Under uniform hypoxia condition H0, the migration speed gradually increased after 8 h to 1.1-fold between 12 h and 16 h, followed by a slight decrease. In contrast, under uniform normoxia condition N, the migration speed of the cancer cells gradually decreased after 8 h to approximately 0.9-fold that in the first 4 h. These changes in migration speed reflect the adaptation of the cells to oxygen tension generated in the device and to a decrease in nutrients in the cell culture medium. Under the intermittent hypoxia condition IH, the migration speed during the first 8 h of normoxia decreased with time. This earlier decrease in migration speed compared to the normoxic condition was possibly caused by device-to-device variation. The period of hypoxia between 8 h and 16 h did not result in any drastic change in migration speed arising from changes in oxygen tension. After returning to normoxic conditions at 16 h, the migration speed of the cells began to increase. The significant difference was confirmed in the steady hypoxic condition H0 compared to the steady normoxic condition N and the intermittent hypoxic condition IH, respectively, by a two-way analysis of variance (ANOVA) test. An increase in MDA-MB-231 cell migration was previously reported under intermittent hypoxia conditions of 1% and 21% O_2_ repeated every 12 h.^22^ The present device can change the oxygen tension in the gel region within 15 min [Figs. 3(d) and 3(e)], which is sufficiently shorter than 4 h during which the average migration speed was evaluated. The migration speed of cancer cells gradually decreased under normoxia but increased under a uniform hypoxic condition. Taken together, similar to cancer cell response to a dynamic chemical gradient,^49^ the present results suggest that MDA-MB-231 cells require several hours to change their migration speed after a change in their surrounding oxygen environment.

The current findings confirm the utility of the present microfluidic device for cellular experiments under conditions of spatiotemporal heterogeneity of oxygen tension. The device is promising for investigating hypoxic microenvironments. Although neither a uniform oxygen tension below 0.3% O_2_ nor an oxygen gradient starting 0% O_2_ could be generated in the present device placed under atmospheric oxygen (21% O_2_) conditions, control of the oxygen concentration surrounding the device should overcome this limitation. This method would facilitate the generation of more physiologically relevant oxygen conditions for future studies such as those in tumor microenvironments with heterogeneous oxygen tension. The effects of hypoxic exposure combined with mechanical and chemical stimuli, such as interstitial flow,^10^ biochemical factors,^45^ and drugs,^50^ will be further investigated with the present microfluidic device. Moreover, studies on the tumor microenvironment while co-culturing different types of cells in the media and gel channels of the present device will also be performed to elucidate the effects of oxygen heterogeneity and cellular oxygen consumption on cell–cell and cell–ECM interactions. The underlying mechanisms in signaling pathways and DNA expression related to enhanced cell activity, triggered by accumulation of hypoxia-inducible factor (HIF),^51^ also remain to be clarified. Interplay between the cells by metabolic substrates and metabolites, which are affected by ion and proton dynamics and pH, need to be elucidated as well.^52^

## CONCLUSIONS

The present double-layer microfluidic device provides superior performance to our previously published system in terms of generating a controlled oxygen level and a rapid response rate, which are indispensable to reproduce the *in vivo* spatiotemporal heterogeneity of oxygen tension. Precise control of oxygen tension to as low as 0.3% O_2_ within 15 min was achieved by optimizing experimental parameters such as medium and gas flow rates and design variables such as the locations of the channels and PC film. The utility of the device for cellular experiments was confirmed by observing the oxygen-dependent behavior of MDA-MB-231 human breast cancer cells. Cell motility was activated under hypoxic conditions, with a local maximum migration speed at 5% O_2_. Under an oxygen gradient, the cells migrated toward an area of mild hypoxia between 5% and 10% O_2_. In addition, intermittent hypoxic exposure revealed that the cells changed their migration speed depending on their surrounding oxygen environment.

## METHODS

### Device design and fabrication

A schematic of the present double-layer microfluidic device is shown in Fig. 1(a). The central channel is 1300 *μ*m wide and contains hydrogel to mimic ECM. This width allows the gel channel to be fully imaged within the field of a 10× objective lens. The flanking media channels are 500 *μ*m wide, having two inlets and three outlets, allowing them to be used as separate media channels or as a connected Y-shaped channel, depending on the experimental setup. Two parallel gas channels 1000 *μ*m wide above the media channels are positioned at a height *H*_g_ from the bottom to allow gas exchange between the other channels during cell culture [Figs. 1(b) and 1(c)]. The distance between the two gas channels was fixed to be the same as the width of the gel channel (1300 *μ*m) to prevent overlapping the gas and gel channels, thus allowing clear observation of the gel channel. The media and gas channels are separated by a PDMS wall. All channels are 150 *μ*m high. The diameter *D* of the microfluidic device is 35 mm, and the thickness *H* is 4 mm. A PC film is embedded inside the device at a height *H*_f_ to prevent oxygen diffusion from the atmosphere. The Cartesian coordinate origin was set at the center of the gel channel, and the *x* and *y*-directions were defined as horizontal normal to and parallel to the gel channel, respectively. The *z*-direction was set to the vertical direction from the bottom of the device.

The microfluidic devices were fabricated with PDMS.^53^ The media and gel channels and the gas channels were patterned on silicon wafers by photolithography. PDMS (Sylgard 184 Silicone Elastomer; The Dow Chemical Co., Midland, MI, USA) was mixed at a 10:1 ratio of base:curing agent, poured over each silicon wafer to a thickness of *H*_g_, and cured in an oven at 65 °C for at least 4 h. The optimal thickness was determined by numerical simulation to be 0.5 mm, as described above. A 32 mm diameter, 0.5 mm-thick PC film with 3 mm port holes punched at the locations of the media, gas, and gel channel ports was positioned on top of the cured PDMS layer of the gas channels. Additional PDMS was then poured over the PC film until the total PDMS layer was 3.5 mm thick, and then the PDMS layer was cured in an oven at 65 °C overnight. The PDMS layer was peeled off the silicon wafers and cut into 35 mm diameter circles. The PDMS layer with the gas channel pattern was punched to form inlets and outlets 2 mm in diameter to allow the infusion of gas mixtures. The channel-patterned surface and the top surface of the other PDMS layer (containing the media and gel channel patterns) were plasma treated (PDC-001; Harrick Plasma Inc., Ithaca, NY, USA) to bond with each other. After incubating the bonded PDMS mold overnight in an oven at 65 °C, 2 mm diameter holes and 1 mm diameter holes were punched to allow access to the media and gel channels, respectively. Finally, the channel-patterned side of the PDMS mold and a glass coverslip were plasma treated and bonded [Fig. 1(d)]. The media and gel channels were coated using a 1 mg/ml poly-D-lysine hydrobromide solution (P7886; Sigma-Aldrich Co. LLC, St. Louis, MO, USA) for better adhesion to the hydrogel. Type I collagen solution (354236; Corning Inc., Corning, NY, USA), adjusted to 1.5 mg/ml at pH 7.4, of approximately 8 *μ*l was injected into the gel channel and polymerized in an incubator (37 °C, 5% CO_2_) for 40 min.^53^ Oxygen sensitive nanoparticles were embedded in the collagen gel as appropriate to validate the oxygen tension in the device. MDA-MB-231 human breast cancer cells were seeded in the collagen gel to demonstrate the utility of the device for cellular experiments.

### Numerical simulation of oxygen tension

Oxygen tension inside the device was computed using commercial finite element software (COMSOL Multiphysics 5.4; COMSOL, Inc., Burlington, MA, USA) by changing the experimental parameters and design variables, such as media and gas flow rates, and the locations of the channels and PC film. The media and gas flows in individual channels were simulated by solving the Navier–Stokes equations coupled with mass continuity for an incompressible fluid,

$$\rho \left(u\cdot \nabla \right)u=\mu \Delta u-\nabla p,$$ (1)

ρ∇·u=0, (2)

where **u** is the velocity vector, *p* is the pressure, *ρ* is the density, and *μ* is the viscosity. The spatial and temporal distribution of oxygen inside the device was then calculated by solving the convection–diffusion equation,

$$\frac{\partial c}{\partial t}=D\Delta c-u\cdot \nabla c,$$ (3)

where *c* is the oxygen concentration, *D* is the diffusion coefficient of oxygen, and *t* is the time. The device was assumed to be in an atmosphere containing 21% O_2_. Medium at 21% O_2_ concentration was supplied using the two inlets of the media channels, while the medium flowed to the middle outlet in the Y-shaped channel. Gas containing 0%–21% O_2_ was supplied to both gas channels to establish a uniform oxygen tension, or gases containing 0% and 21% O_2_ were supplied to the left-hand and right-hand side gas channels to generate an oxygen gradient [see Fig. 1(c)], respectively. Zero pressure and convection flux conditions were set at the outlets of the media and gas channels, and a no-slip condition was applied on the channel walls for fluid flow analysis. Boundary conditions for the oxygen concentration were set according to Henry's law.^40,54^ The oxygen concentration at the interfaces between PDMS and the gas phase (atmosphere and gas mixture in the gas channel) was set corresponding to the product of the solubility coefficient of oxygen in PDMS and the partial pressure of oxygen. At the interfaces between PDMS and media or gel, a partition condition was applied, which balanced the mass flux of oxygen to satisfy the continuity of partial pressure of oxygen,

$$\frac{c_{\mathrm{PDMS}}}{S_{\mathrm{PDMS}}}=\frac{c_{\mathrm{channel}}}{S_{\mathrm{channel}}},$$ (4)

where *c*_PDMS_ and *S*_PDMS_ are the oxygen concentration and the solubility of oxygen in PDMS, respectively, and *c*_channel_ and *S*_channel_ are those in the media and gel channels. Oxygen consumption by cells cultured in the device was not considered to investigate oxygen controllability of the microfluidic device itself. Although the cells cultured in the device consume oxygen and might cause local spatial oxygen gradients, the contribution to the oxygen concentration due to oxygen consumption by the cells could be considered to be small since the cell density was relatively low in the present study.^55^ The initial condition of oxygen concentration in each material was set to 21% O_2_. The parameter values used for density, viscosity, oxygen diffusivity, and oxygen solubility for the medium, gas, gel, PDMS, and PC film are summarized in Table I.^42,54^ The solubilities of oxygen in PDMS and the PC film were assumed to be the same since they are reportedly within the same range.^56,57^ The medium and gas flow rates were changed based on the Péclet number *Pe*, which characterizes mass transport by the ratio of advection to diffusion. The values of *Pe* for the medium and gas flows (*Pe*_m_, *Pe*_g_) were 0, 1, 10, 100, and 500 (Table I). The locations (*H*_g_, *H*_f_) of the gas channels and PC film from the bottom were changed from (0.25 mm, 0.5 mm) to (1.5 mm, 3 mm) while keeping the device diameter *D* and height *H* at 35 mm and 4 mm, with *H*_f_ =2*H*_g_ [see Fig. 1(b)]. The computational models consisted of approximately 870 000–1 250 000 computational elements.

**TABLE I.** Physical properties of each component and parameters for numerical simulations.

	Medium	Gas	Gel	PDMS	PC film
Density, ρ (kg/m^3^)	1.0 × 10^3^	1	1.0 × 10^3^		
Viscosity, *μ* (Pa s)	1.0 × 10^–3^	1.0 × 10^–5^			
Diffusivity of oxygen, *D* (m^2^/s)	2.0 × 10^–9^	2.0 × 10^–5^	2.0 × 10^–9^	4.0 × 10^–9^	2.0 × 10^–12^
Solubility of oxygen, *S* (mM/atm)	0.218		0.218	1.25	1.25
Péclet number, *Pe*	0–500	1–500			
Average velocity, *U* (m/s)	0–2.0 × 10^–3^	0.02–10			
Flow volume, *Q* (ml/min)	0–9.0 × 10^–3^	0.18–90			
Oxygen tension, *c* (%)	21	0–21			

### Measurement of oxygen tension

Oxygen tension generated inside the microfluidic device was measured using a phosphorescent nanoparticle-based oxygen sensitive probe (MitoImage MM2 Probe; Luxcel Biosciences Ltd., Cork, Ireland).^58^ These particles exhibit blue fluorescence at 430–450 nm and red phosphorescence at 640–670 nm when excited with 390–405 nm ultraviolet light. The fluorescence intensity was unaffected by oxygen tension. In contrast, phosphorescence was dependent on the oxygen tension, and thus, its intensity was used to quantify oxygen tension. The oxygen-sensitive nanoparticles were mixed with type I collagen gel at 100 *μ*g/ml and embedded in the gel channel of the microfluidic device by following the gel filling procedure described above. The microfluidic device was then set in a stage incubator (INUBSF-ZILCS; Tokai Hit Co., Ltd., Shizuoka, Japan) (37 °C) mounted on a spinning disk confocal microscope (IX83-DSU; Olympus Corporation, Tokyo, Japan).

We tested the oxygen sensitivity of the nanoparticles embedded in each microfluidic device by filling the stage incubator with a gas mixture containing a defined percentage of O_2_ and 5% CO_2_ balanced with N_2_ at 200 ml/min. The oxygen concentration in the stage incubator was assured to be the same as that in the infused gas mixture by using a pocket oxygen monitor (OX-03, Riken Keiki, Co., Ltd., Tokyo., Japan). The same gas mixture was simultaneously introduced into the microfluidic gas channels at 18 ml/min. We waited for at least 30 min before capturing the fluorescence and phosphorescence of the oxygen-sensitive nanoparticles with the microscope to allow thermal equilibrium and stabilization of the oxygen tension inside the device. This procedure was conducted by supplying gas mixtures with at least three different oxygen concentrations, including 21% and 0% O_2_, to obtain the relationship between the oxygen concentration and phosphorescence intensity.

Various steady-state oxygen conditions were then generated by supplying gas mixtures to the gas channels and the stage incubator, as summarized in Table II. A normoxic condition (N) was generated by supplying a gas mixture consisting of 21% O_2_, 5% CO_2_, and 74% N_2_ to both gas channels and the stage incubator. Uniform hypoxic conditions (H0–H10) were generated by supplying gas mixtures of 0%–10% O_2_ and 5% CO_2_ balanced with N_2_ to the gas channels. An oxygen gradient (G) was generated by supplying two different gas mixtures (21% O_2_, 5% CO_2_, and 74% N_2_, and 0% O_2_, 5% CO_2_, and 95% N_2_) to each of the two gas channels. In the conditions of H0–H10 and G, the stage incubator was filled with a gas mixture consisting of 21% O_2_, 5% CO_2_, and 74% N_2_. Finally, an anoxic condition (A) was generated by supplying a gas mixture consisting of 5% CO_2_ and 95% N_2_ to both gas channels and the stage incubator. The flow rate of the gas mixture into the gas channel was set to 18 ml/min (*Pe*_g_ = 100), while a flow rate of 200 ml/min was supplied to the stage incubator. Conversely, medium was maintained under static conditions in the media channels during experimental validation of the oxygen tension. For each oxygen condition, fluorescence and phosphorescence images of the oxygen-sensitive nanoparticles were captured after supplying the gas mixtures for more than 30 min.

**TABLE II.** Oxygen concentration in the gas mixture supplied to the gas channels and the stage incubator to generate each oxygen condition in the validation experiment (%).

Condition	Gas channel						Stage incubator		
	Left			Right					
	O_2_	CO_2_	N_2_	O_2_	CO_2_	N_2_	O_2_	CO_2_	N_2_
Normoxia (N)	21	5	74	21	5	74	21	5	74
Hypoxia (H0–H10)	0–10	5	95–85	0–10	5	95–85	21	5	74
Oxygen gradient (G)	0	5	95	21	5	74	21	5	74
Oxygen gradient 2 (G2)	0	5	95	10	5	85	21	5	74
Anoxia (A)	0	5	95	0	5	95	0	5	95

The transient state of the oxygen condition in the microfluidic device was measured by time-lapse imaging of the fluorescence and phosphorescence of the oxygen-sensitive nanoparticles. Oxygen conditions were changed by switching the gas mixtures supplied to the gas channels; i.e., from the normoxic condition (N), generated using the 21% O_2_ gas mixture to the hypoxic condition (H0), generated using the 0% O_2_ gas mixture, and vice versa. Microscope images were recorded every minute for the first 3 min, followed by every five minutes to 20 min, and at 30 min, 60 min, 3 h, and 6 h after changing the supplied gas mixture.

The fluorescence and phosphorescence intensities were quantified using open-source image processing software (ImageJ; NIH, Bethesda, MD, USA). The fluorescence and phosphorescence intensity profiles *I*_F_ and *I*_P_ were quantified by setting a rectangular region of interest (ROI) of 1024 × 50 pixels (1331 *μ*m × 65 *μ*m) across the gel channel. The ROI was divided into small sections of 100 *μ*m × 65 *μ*m inside the gel channel, and the space-averaged fluorescence and phosphorescence intensities $\bar{I}$_F_(*x*) and $\bar{I}$_P_(*x*) in each section were obtained. In order to eliminate the effects of light source fluctuations, the phosphorescence intensity $\bar{I}$_P_(*x*) under each condition was normalized by the fluorescence intensities $\bar{I}$_F_(*x*) at the same time point and $\bar{I}$_F0_(*x*) at the first time point in the experiment: ${\bar{I}}^{\prime }$(*x*) = $\bar{I}$_P_(*x*) × $\bar{I}$_F0_(*x*)/$\bar{I}$_F_(*x*). Then, the normalized phosphorescence intensity ${\bar{I}}^{\prime }$(*x*) was converted to an oxygen concentration, *c*(*x*), by substituting ${\bar{I}}^{\prime }$(*x*) into the Stern–Volmer equation as follows:

$$c\left(x\right)=\frac{\left(\frac{{{\bar{I}}^{\prime }}_{A}\left(x\right)-{{\bar{I}}^{\prime }}_{\mathrm{BG}}\left(x\right)}{{\bar{I}}^{\prime }\left(x\right)-{{\bar{I}}^{\prime }}_{\mathrm{BG}}\left(x\right)}-1\right)}{K_{q}\left(x\right)}\times 100,$$ (5)

where ${\bar{I}}^{\prime }$_A_(*x*) represents the normalized phosphorescence intensity under anoxia, ${\bar{I}}^{\prime }$_BG_(*x*) is the background noise in the normalized phosphorescence caused by device materials (PDMS or polycarbonate film) and cell culture media, and *K*_q_(*x*) is the quenching constant. The values of ${\bar{I}}^{\prime }$_BG_(*x*) and *K*_q_(*x*) were derived so as to best fit the intensity data and the oxygen concentrations estimated from the aforementioned oxygen sensitivity test of nanoparticles to the Stern–Volmer equation.

The validation experiments were conducted with three devices for each oxygen condition in both steady and transient states.

### Cellular experiments under controlled oxygen tension

Feasibility of the microfluidic device for cellular experiments was evaluated by observing the migrational behavior of cancer cells under controlled oxygen tension inside the device. A Green Fluorescent Protein (GFP)-labeled MDA-MB-231 human breast cancer cell line (AKR-201; Cell Biolabs, Inc., San Diego, CA, USA) was passaged in Dulbecco's Modified Eagle's Medium (DMEM) (D5671; Sigma-Aldrich) supplemented with 10% fetal bovine serum (FBS) (F7524; Sigma-Aldrich) and 1% L–Glutamine–Penicillin–Streptomycin solution (G6784; Sigma-Aldrich) (DMEM/FBS) and cultured in a humidified incubator (37 °C, 5% CO_2_). Cells were collected by trypsinization and mixed with 1.5 mg/ml type I collagen solution prepared, as described above for the gel filling procedure, to a final cell density of 2 × 10^5^ cells/ml. After introducing the cell-mixed collagen solution into the gel channel, the microfluidic device was rotated upside down for less than 2 min to distribute the cells three-dimensionally by gravity. The device was then placed in a humidified incubator (37 °C, 5% CO_2_) for 40 min to polymerize the collagen gel, followed by supplying DMEM/FBS medium supplemented with 50 ng/ml epidermal growth factor (AF-100–15; PeproTech, Inc., Rocky Hill, NJ, USA) into the media channels. After 24 h in the incubator, the microfluidic device was set in a stage incubator (37 °C, 5% CO_2_) on a spinning disk confocal microscope. Ethics approval is not required for this study because no animal or human experiments were conducted by the authors.

A uniform hypoxic condition (H0**–**H10) or normoxic condition (N) was generated by supplying a humidified gas mixture containing 0, 1, 3, 5, 10, or 21% O_2_ and 5% CO_2_ balanced with N_2_ at a flow rate of 18 ml/min to the gas channels (see Table II). An oxygen gradient (G) was generated by supplying two humidified gas mixtures comprising 21% O_2_, 5% CO_2_, 74% N_2_, and 5% CO_2_, 95% N_2_ to the different gas channels. In addition, the cells were exposed to an intermittent hypoxic condition (IH) by alternatively supplying gas mixtures containing 21% O_2_ and 0% O_2_ to the gas channels at 8 h intervals. For simplicity, medium was not introduced to the media channels during the cell culture experiment, thus keeping the medium at a constant static condition. After allowing thermal equilibrium of the device (more than 30 min), time-lapse imaging of the migrating cancer cells was conducted every 10 min for 24 h. At each time point, confocal microscope images of the GFP-labeled cells in the collagen gel were taken at 2.1 *μ*m intervals in the *z*-direction (approximately 72 images) and one phase-contrast microscope image of the middle horizontal cross section of the gel channel was obtained. Finally, cell viability was assessed using a Live/Dead assay kit (R37601; Thermo Fisher Scientific Inc., Waltham, MA, USA).

Three-dimensional migration of the cells was tracked using commercial image analysis software (MetaMorph; Molecular Devices, LLC, San Jose, CA, USA). The distance migrated by each cell was measured every 10 min, and the migration speed at each time point was calculated. Live cells were counted at the start and end point of each experiment to quantify cell proliferation. The cellular experiments were conducted with three or more devices for each oxygen condition, and the migration speeds of 30–100 cells were measured at every time point in each device. Kruskal–Wallis tests followed by Dunn's post-hoc tests for multiple comparisons were performed to compare migration speeds for cells under each uniform oxygen tension condition. Furthermore, Welch's *t* tests were performed to compare proliferation rates observed under the different oxygen conditions. Two-way analysis of variance (ANOVA) tests were performed to compare the variations of migration speed in the different conditions. Statistical significance was inferred for *p *<* *0.05.

## SUPPLEMENTARY MATERIAL

See the supplementary material for the details of computational analysis and measurement of cancer cell migration.
